# Supplementary material for: Development of a Set of Indicators for Measuring and Improving Quality of Rehabilitation Care after Ischemic Stroke
Source: Healthcare (Basel). 2023 Jul 19;11(14):2065. doi: 10.3390/healthcare11142065 (PMC10378746; doi:10.3390/healthcare11142065)
Supplement: Supplementary file 1 [file healthcare-11-02065-s001.zip › healthcare-2478461-supplementary.pdf]

Development of a Set of Indicators for Measuring and Improving Quality of Rehabilitation Care after Ischemic Stroke

Maria Cristina De Cola [1], Augusto Ielo [1,*], Francesco Corallo[1], Sebastiano Pollina Addario [2], Salvatore Scondotto [2], Alessandra Allotta [2], Giovanna Fantaci [2], Placido Bramanti [1,3], Rosella Ciurleo [1]

1. IRCCS Centro Neurolesi Bonino-Pulejo, 98124 Messina, Italy
2. Assessorato della Salute, Dipartimento Attività Sanitarie e Osservatorio Epidemiologico, 90145 Palermo, Italy
3. Faculty of Psychology, Università Degli Studi eCampus, Via Isimbardi 10, 22060 Novedrate, Italy

* Correspondence: augusto.ielo@irccsme.i

Supplementary material

1. Quality indicators formulas

1.1 Number of Rehabilitation Admissions (NoRA)

The NoRA indicator quantifies the number of rehabilitation admissions for ischemic stroke, and is calculated as

$$NoRA = \sum_{j=1}^{n} d_j$$

$$d_j \in \{0,1\}, j = 1, \dots, n.$$

Where n is the total number of hospital's discharges in a given period of time, j indexes the hospital's discharges, and d is a 0/1 variable taking the value 1 if discharge j is eligible to be used in the indicator or 0 otherwise.

1.2 Average Waiting Time for Rehabilitation (AWTR)

The AWTR is the average waiting time for rehabilitation admission following the discharge from acute hospitalization for ischemic stroke. AWTR indicator is calculated as

$$AWTR = \frac{\sum_{j=1}^{n} t_j \cdot d_j}{\sum_{j=1}^{n} d_j}$$

$$d_j \in \{0, 1\}, t_j \in \{0, 1, 2, \dots\}, j = 1, \dots, n.$$

Where n is the total number of hospital's discharges in a given period of time, j indexes the hospital's discharges, d is a 0/1 variable taking the value 1 if discharge j meets the criteria for cohort eligibility or 0

otherwise, and *t* is the time in days between the discharge from acute care and the admission to rehabilitation.

1.3 Home Discharges (HD)

The HD indicator is the proportion of discharges to the patient's home following the admission to rehabilitation for ischemic stroke. HD indicator is calculated as

$$HD = \frac{\sum_{j=1}^{n} h_j \cdot d_j}{\sum_{j=1}^{n} d_j}$$

$$d_j \in \{0,1\}, h_j \in \{0,1\}, j = 1, \dots, n.$$

Where *n* is the total number of hospital’s discharges in a given period of time, *j* indexes the hospital’s discharges, *d* is a 0/1 variable taking the value 1 if discharge *j* meets the criteria for cohort eligibility or 0 otherwise, and *h* is a 0/1 variable taking the value 1 if the patient is discharged to his or her home, 0 otherwise.

1.4 Protected Discharges (PD)

The PD indicator is the proportion of protected discharges following the admission to rehabilitation for ischemic stroke. PD indicator is calculated as

$$PD = \frac{\sum_{j=1}^{n} p_j \cdot d_j}{\sum_{j=1}^{n} d_j}$$

$$d_j \in \{0,1\}, p_j \in \{0,1\}, j = 1, \dots, n.$$

Where *n* is the total number of hospital’s discharges in a given period of time, *j* indexes the hospital’s discharges, *d* is a 0/1 variable taking the value 1 if discharge *j* meets the criteria for cohort eligibility or 0 otherwise, and *p* is a 0/1 variable taking the value 1 if the patient underwent a protected discharge.

1.5 Acute Discharges (AD)

The HD indicator is the proportion of discharges to the patient's home following the admission to rehabilitation for ischemic stroke. HD indicator is calculated as

$$AD = \frac{\sum_{j=1}^{n} a_j \cdot d_j}{\sum_{j=1}^{n} d_j}$$

$$d_j \in \{0,1\}, a_j \in \{0,1\}, j = 1, \dots, n.$$

Where *n* is the total number of hospital’s discharges in a given period of time, *j* indexes the hospital’s discharges, *d* is a 0/1 variable taking the value 1 if discharge *j* meets the criteria for cohort eligibility or 0 otherwise, and *a* is a 0/1 variable taking the value 1 if the patient has been transferred to an acute ward, 0 otherwise.

1.6 Average Change in Level of Disability (ACLoD)

The ACLoD indicator quantify the average change in the level of disability from admission to discharge. ACLoD indicator is calculated as

$$MCR = \frac{\sum_{j=1}^{n}(s_{2j} - s_{1j}) \cdot d_j}{\sum_{j=1}^{n} d_j}$$

$$d_j \in \{0, 1\}, s_1, s_2, j = 1, \dots, n.$$

Where *n* is the total number of hospital's discharges in a given period of time, *j* indexes the hospital's discharges, *d* is a 0/1 variable taking the value 1 if discharge *j* meets the criteria for cohort eligibility or 0 otherwise, s1 is the Barthel Index at admission and s2 is the Barthel Index score at discharge [41].

1.7 Rehabilitation Goals Achievement (RGA)

The RGA indicator is the proportion of rehabilitation hospitalization in which rehabilitation goals were achieved. RGA indicator is calculated as

$$RGA = \frac{\sum_{j=1}^{n} g_j \cdot d_j}{\sum_{j=1}^{n} d_j}$$

$$d_j \in \{0, 1\}, g_j \in \{0, 1\}, j = 1, \dots, n.$$

Where *n* is the total number of hospital's discharges in a given period of time, *j* indexes the hospital's discharges, *d* is a 0/1 variable taking the value 1 if discharge *j* meets the criteria for cohort eligibility or 0 otherwise, and *g* is a 0/1 variable taking the value 1 if the rehabilitation goal was achieved or 0 otherwise.
